# Interactive multiobjective optimization for finding the most preferred exercise therapy modality in knee osteoarthritis

**DOI:** 10.1080/07853890.2021.2024876

**Published:** 2022-01-13

**Authors:** Babooshka Shavazipour, Bekir Afsar, Juhani Multanen, Kaisa Miettinen, Urho M. Kujala

**Affiliations:** a Faculty of Information Technology, University of Jyvaskyla, Jyvaskyla, Finland; b Faculty of Sport and Health Sciences, University of Jyvaskyla, Jyvaskyla, Finland; c Department of Physical Medicine and Rehabilitation, Central Finland Central Hospital, Jyvaskyla, Finland

**Keywords:** Knee osteoarthritis, cost-effective exercise therapy modality, pain, physical function, decision making, decision support

## Abstract

**Background:**

There are no explicit guidelines or tools available to support clinicians in selecting exercise therapy modalities according to the characteristics of individual patients despite the apparent need.

**Objective:**

This study develops a methodology based on a novel multiobjective optimization model and examines its feasibility as a decision support tool to support healthcare professionals in comparing different modalities and identifying the most preferred one based on a patient’s needs.

**Methods:**

Thirty-one exercise therapy modalities were considered from 21 randomized controlled trials. A novel interactive multiobjective optimization model was designed to characterize the efficacy of an exercise therapy modality based on five objectives: minimizing cost, maximizing pain reduction, maximizing disability improvement, minimizing the number of supervised sessions, and minimizing the length of the treatment period. An interactive model incorporates clinicians’ preferences in finding the most preferred exercise therapy modality for each need. Multiobjective optimization methods are mathematical algorithms designed to identify the optimal balance between multiple conflicting objectives among available solutions/alternatives. They explicitly evaluate the conflicting objectives and support decision-makers in identifying the best balance. An experienced research-oriented physiotherapist was involved as a decision-maker in the interactive solution process testing the proposed decision support tool.

**Results:**

The proposed methodology design and interactive process of the tool, including preference information, graphs, and exercise suggestions following the preferences, can help clinicians to find the most preferred exercise therapy modality based on a patient’s needs and health status; paving the way to individualize recommendations.

**Conclusions:**

We examined the feasibility of our decision support tool using an interactive multiobjective optimization method designed to help clinicians balance between conflicting objectives to find the most preferred exercise therapy modality for patients with knee osteoarthritis. The proposed methodology is generic enough to be applied in any field of medical and healthcare settings, where several alternative treatment options exist.

**KEY MESSAGES**
- We demonstrate the potential of applying Interactive multiobjective optimization methods in a decision support tool to help clinicians compare different exercise therapy modalities and identify the most preferred one based on a patient’s needs.
- The usability of the proposed decision support tool is tested and demonstrated in prescribing exercise therapy modalities to treat knee osteoarthritis patients.

## Introduction

Osteoarthritis (OA) is the most common form of arthritis [1] and a leading source of chronic pain and disability worldwide [2]. Knee OA, in particular, causes a heavy burden to the population, as pain and stiffness in this large weight-bearing joint often lead to significant disability requiring surgical interventions. The prevalence of radiographic knee OA varies between 4 and 79%, depending on the age category, country of origin, and sex distribution of the study population [3]. Overall, the prevalence of knee OA increases with age [4, 44]. The high prevalence of knee OA and the predicted aging of the population will accentuate the burden of knee OA on health care systems. While no good structure modifying drugs are available to prevent or treat OA, exercise therapy is an important means of controlling OA-induced pain and loss of function.

Previous meta-analyses of randomized controlled trials (RCTs) on the effects of exercise therapy modality in the treatment of patients with knee OA show that exercise therapy modality improves physical function [,^7^, ^8^], reduces pain [,^7^, ^8^], eases depression [5], decreases anxiety [6], and improves the quality of life [7,8]. Given that exercise adherence typically declines over time, supervised therapies are expected to provide better results than non-supervised exercise therapy modalities in some diseases. Some studies also confirm the superiority of supervised exercises on pain reduction and disability improvement for patients with knee OA [9]. However, supervised training is more expensive than non-supervised training, representing a conflict between cost and recovery. Furthermore, previous studies [9] found no differences in effects between different types of exercise therapy modality (or support for individualization of exercise therapy modalities) based on studies where individual baseline characteristics were not used in tailoring the exercise therapy modality. There are no explicit guidelines or tools available to support clinicians in tailoring the exercise therapy modalities to individual patients based on their characteristics. More research is needed to understand better which type of exercise therapy modality is most suitable for improving physical function and reducing pain while keeping costs acceptable, taking individual characteristics into account.

Multiobjective optimization methods are designed to support a domain expert referred to as a decision-maker in identifying the best balance between conflicting objectives (e.g. [10–13]). Depending on whether lower or higher values are desirable for the characteristic represented by an objective, we either minimize or maximize it, respectively. Thus, we, e.g. minimize cost and maximize physical functionality. Support is needed when they are conflicting (e.g. no improvement in physical functionality is possible without an extra cost) since there exists no well-defined best or optimal solution but several compromises, with different trade-offs. Nonetheless, eventually, only a single solution must be chosen for implementation. Therefore, the decision-maker with domain expertise plays a vital role in finding the most preferred solution by providing his/her preferences. Interactive methods enable the decision-maker to iteratively provide preference information, modify preferences if needed, and learn about interdependencies among the objectives and the feasibility of the preferences [14]. Indeed, the decision-maker is actively involved during the solution process, giving them a chance of modifications based on their new insight and available options. This iterative interaction will also reduce the cognitive load set of comparing a long list of alternatives at a time and let the decision-maker concentrate on a subset of optimal options based on their preferences. This also decreases the time of analysis and comparisons and grows the confidence of the decision-maker during the iterative solution process. These reasons make interactive methods the best option for clinical analysis that needs expert knowledge and careful comparisons.

This paper introduces a novel interactive multiobjective optimization method incorporating decision-makers’ preferences to be used in a decision support tool. It is inspired by the NIMBUS method [15], which generates a group of compromise solutions (satisfying the preferences as well as possible) to be shown to the decision-maker in each iteration. Here, a decision-maker refers to a clinician, physiotherapist, or some other healthcare professional, and solutions refer to exercise therapy modalities. In the set of compromise solutions, none of the conflicting objectives characterizing therapies can be improved without impairing at least one of the others. Based on a patient’s needs and health status, the decision-maker will choose and prescribe the most preferred exercise therapy modality from the set of compromises identified by the proposed methodology.

To the best of our knowledge, this study is the first application of multiobjective optimization methods to support decision-making and treatment analysis in OA. Multiobjective optimization has been applied in healthcare-related problems (e.g. in different aspects of medical management and technologies, such as emergency management systems [16,17], scheduling [18], transport and logistics [19], resource and location-allocation [20,21], patient allocation [22], radiation therapy [23], and brachytherapy [24]).

This study develops methodology and demonstrates the feasibility of a decision support tool for comparing different exercise therapy modalities in knee OA as a proof of concept. Therefore, it can be adjusted for different (numbers of) exercise therapy modalities and various objectives or be applied to compare the efficacy of any other treatment in other diseases (assuming appropriate data are available).

This methodological study is the first step in a three-step process of developing a straightforward way to choose a personalized optimal exercise therapy modality for each patient based on the available research data. Here we develop theory and test how to use the proposed methodology and decision support tool in a simple example as a proof of concept. The next steps will be a corresponding analysis using more detailed individual data from many trials and then an easy user interface for decision-makers. To proceed towards this much-needed personalized medicine supporting aid, we need to proceed step by step. We have started the process by introducing the methodology in this study. This novelty can be counted as the new wave of digitalization and data analytics that connect researchers from different disciplines to make the best use of data and improve traditional methods to select intervention types that should be most beneficial and cost-effective for each patient.

## Methods

As mentioned earlier, the paramount aim of this paper is to support decision-makers in studying, analyzing, and comparing available exercise therapy modalities by considering various (conflicting) objectives simultaneously. The decision-maker of this study has a Ph.D. in Physiotherapy with experience in conducting knee OA studies. Besides a decision-maker, the design involves an analyst, whose responsibility is generating information, modeling, identifying the suitable method, and performing all mathematical calculations. An analyst can be a human, a computer program, or a combination. In this study, a group of analysts was involved, including a university professor (with more than 30 years of research experience) and two post-doctoral researchers (with more than 10 years of research experience). Also, we had set up a face-to-face meeting to conduct the experiment with our decision-maker. All the authors were present in the experiment.

Information on different exercise therapy modalities can be collected from previous meta-analyses of RCTs. These data have to be pre-processed based on clinical objectives that are set by a decision-maker. Based on this information, a relevant multiobjective optimization problem can be formulated. We propose a novel interactive multiobjective optimization method to support the decision-maker in finding the compromise solutions that best reflect the decision-maker’s preference information (see, e.g. [11,14,25] and references therein for the basic features of interactive methods). Hence, the decision-maker augments the available data with one’s domain expertise, iteratively provides one’s preference information, and sees what kinds of therapies reflect the preferences best and what kinds of trade-offs exist. At the same time, the iterative nature reduces the cognitive load since the decision-maker can concentrate on therapies that satisfy the preferences best.

Furthermore, the decision-maker can modify the preferences based on the insight gained and eventually identify the most preferred exercise therapy modality considering the patient’s needs and health status. One should note that we are not providing a global answer or recommendation. The decision-maker can analyze the suggested therapies using the proposed methodology and prescribe the most appropriate one based on recent clinical guidelines and individual patient characteristics.

**Figure 1.**
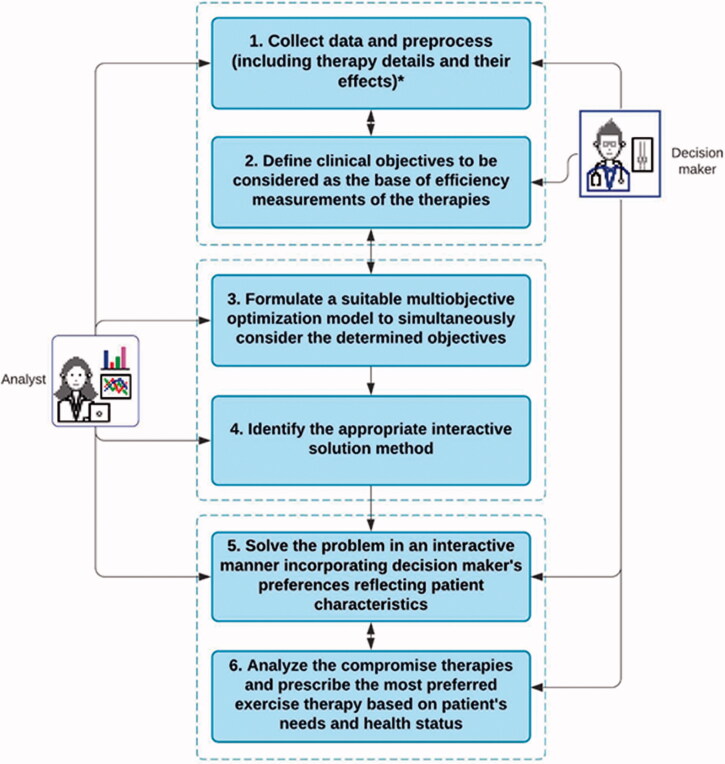
Proposed methodology for decision support tool in knee OA. *For example, types of exercise, cost, changes in pain and function, number and duration of the exercise session.

Figure 1 describes an overall view of designing the proposed decision support tool for finding the most preferred exercise therapy modality in knee OA, where the decision-maker and an analyst participate. The proposed methodology includes six phases, each of which is numbered and shown in the boxes in the figure. These phases of the proposed methodology are described as follows.

### Inclusion criteria and data extraction (phases 1 and 2)

As a starting point, we considered the RCTs selected by Goh et al. [8] that evaluated the efficacy of exercise therapy modalities in knee OA, hip OA, and knee and hip OA. The authors [8] conducted a systematic literature search from “*the dates of inception*” to December 2017. They included papers reporting trials comparing an exercise intervention and a non-exercise one in the knee and hip OA treatment. They also established specific eligibility criteria after the literature search. They included trials if participants (i) had not undergone knee or hip joint replacement surgery, (ii) had only exercise therapy modality without additional treatment, and (iii) were assigned to usual care in the control group. The participants in the control groups received usual care, which may include advice and instructions, physiotherapy, home exercises, or being on a waiting list. The reported outcomes were pain, function, performance, and quality of life (QoL).

**Figure 2.**
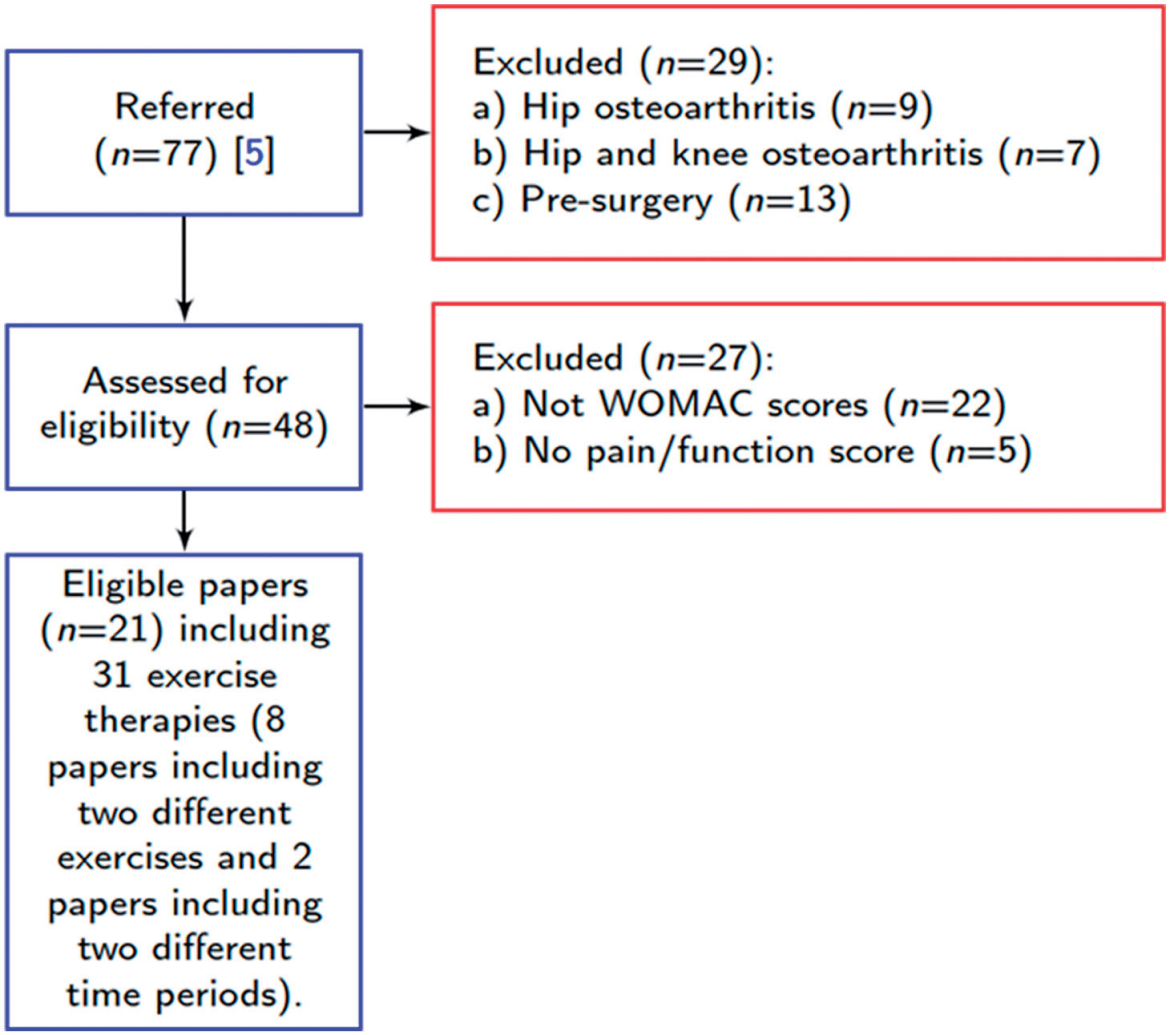
Selection of papers for data collection. WOMAC: Western Ontario and McMaster Universities.

Goh et al. [8] selected 77 RCTs for the meta-analysis based on a literature search and the specific eligibility criteria mentioned above. We used the same inclusion criteria for the exercise therapy studies as in Goh et al. [8], except that we included studies on patients with knee OA only and where the data of the Western Ontario and McMaster Universities (WOMAC) scale had been used as an outcome measure for pain and function. Furthermore, we ruled out studies on patients who underwent an exercise therapy program before knee replacement surgery. The WOMAC scale was chosen as it is recommended and most commonly used as a disease-specific outcome instrument in patients with OA [26]. By contrast, the Knee Injury and Osteoarthritis Outcome Score (KOOS) is intended to be used mainly for knee injuries that can result from various reasons, including OA. In addition, WOMAC is the most frequently used measure reported in previous studies. Therefore, because we did not have access to the individual data but only the mean and variance of each group, we had to exclude studies that did not report WOMAC scores for pain and function. This is because extracting or converting other measures to WOMAC without individual data causes some errors, making the converted ones unreliable. Preoperative exercise programs were excluded because people waiting for knee replacement surgeries often have mobility restrictions due to pain and disabilities, and we do not know how much physical activity is safe and feasible for people with severe knee OA [27]. Moreover, several testing types and results were listed for performance outcomes. This heterogeneity makes a quantitative comparison between the outcomes of different therapies challenging. In addition, QoL outcomes were not measured in a majority of the RCTs (33 papers). Therefore, in this paper, we did not consider outcomes for performance and QoL measurements. Figure 2 summarizes the papers (and therapies) meeting our inclusion criteria which were the following (i) participants had only knee OA, (ii) participants had no surgery before the exercise therapy modality, and (iii) outcomes were reported for pain and function in the WOMAC scale.

According to the reasoning above, we collected data from the selected papers. Out of the 77 RCTs identified by Goh et al. [8], papers not following the above-mentioned inclusion criteria were excluded. As a result, the required data were collected from the remaining 21 papers (including 31 different interventions) and recorded in Table 1 in Supplementary Material A. We listed the therapies, the outcomes for pain and function in the WOMAC scale, the number of supervised sessions, and the therapies’ lengths. We also adjusted the ranges of the WOMAC scale to the same range (0–20 for pain, 0–68 for function) if the reported outcomes had different ranges for the WOMAC scale.

### Multiobjective optimization for knee OA (phase 3)

As previously mentioned, the focus of this study is on finding the most preferred exercise therapy modality for a patient with knee OA. To characterize the goodness of an exercise therapy modality, we considered five conflicting objectives (based on the data availability and the decision-maker’s assessment): minimizing cost, maximizing pain reduction and function improvement, minimizing the number of supervised exercise sessions, and the length of the treatment period. However, a decision-maker could select different objectives to be considered in the proposed decision support tool. We wanted to optimize all of these objectives simultaneously. As they are conflicting, a therapy that can have the best performance in all objectives does not exist. However, compromises with trade-offs exist, as mentioned in the Introduction. With the aid of multiobjective optimization methods [11], we can support the decision-maker to identify the most preferred exercise therapy modality among the compromises and then make the final choice based on the patient’s characteristics. In what follows, we discuss the objectives in more detail.

#### Minimize the cost of therapy

We estimated the cost of each exercise therapy modality based on personal expenses (i.e. patient elements of the price), such as the number of supervised or unsupervised training sessions, length of each session, number of trainees in each group, types of equipment, and possible checkpoint calls. Moreover, costs were measured from the time between the baseline and the end-point of the outcome measure (later follow-ups are not included). Costs were estimated with the current prices (early 2021) in Finland. However, costs can simply be adapted for any time in any other country. For cost estimations, two authors with a long-term clinical background checked all the selected papers; included interventions in detail; and, in a few cases, contacted the authors of the papers for further information. Table 1 describes various basic individual elements of costs utilized in the cost estimation in this study. More details of cost estimation for exercise therapy modalities considered in this study can be found in Supplementary Material D.

**Table 1.** Basic individual costs are utilized in the cost estimation.

Fee for physiotherapy (or similar exercise instructor)	
Individual 1 h	100 €
Individual 45 min	75 €
Individual 30 min	50 €
Individual (group 1/5) 1 h	20 €
Individual (group 1/5) 45 min	15 €
Individual (group 1/5) 30 min	10 €
Non-supervised training: the rehabilitation centre, gym, etc.	
Individual/session	10 €
Non-supervised training: home	
Individual/session	0 €
Phone call/SMS message, etc.	10 €
Exercise equipment	0 €

#### Maximize pain reduction

As previously mentioned, we only had average values of WOMAC scores for pain reduction. We did not have individual data for the patients in the exercise and control groups in each exercise therapy modality. Therefore, we considered the differences between the mean of the WOMAC pain scores pre- and post-intervention as the pain reduction. Furthermore, to take into account the control groups and to be able to measure clinically meaningful change or improvement, we considered the expected net change in pain as the pain reduction objective. We defined the net change as the difference between the mean change in the exercise and control groups.

#### Maximize improvement in physical function

Like pain, we considered the expected net change in WOMAC score for physical function as the disability improvement objective.

#### Minimize the number of supervised training sessions

Organizing supervised training sessions is always challenging due to the limited resources available. Therefore, for the fourth objective, we minimized the number of supervised training sessions.

#### Minimize the length of treatment

Finally, the fifth objective is minimizing the length of treatment, which is often a concern for patients and healthcare professionals.

#### Proposed multiobjective optimization problem

With the objectives discussed so far, we formulated a multiobjective optimization problem to support decision-making and analysis of different therapies to determine the most preferred one. Mathematical formulations are provided in Equation (1) in Supplementary Material B.

Minimize the cost of therapy

Maximize expected net improvement in pain reduction

Maximize expected net improvement in physical function

Minimize the number of supervised training sessions

Minimize the length of treatment

Subject to one therapy is selected from a list of options.

### Proposed interactive method (phases 4–6)

This section proposes a new interactive multiobjective optimization method to be applied to solve the problem formulated. The interaction means that the decision-maker’s preferences are taken iteratively into account in the solution process in finding the most preferred therapy. The proposed interactive method is inspired by the NIMBUS method [15], in which multiple solutions reflecting the preferences as well as possible are generated and shown to the decision-maker in each iteration. However, in NIMBUS, the decision-maker’s preferences are expressed by classifying the objectives into pre-defined classes, which is not the case in this study. Our method differs from NIMBUS in two perspectives, namely, (i) preference type and (ii) way of showing solutions to the decision-maker.

As preferences, the decision-maker can provide desirable upper and lower bounds for the possible outcomes of five objectives (also called objective values). These values are meaningful and understandable for the decision-maker. The decision-maker can conveniently follow how feasible the desires were when one sees the optimization results, given that all information is about objective values, and no cognitive mappings are needed. These bounds form a so-called preferred range. A lower bound is the preferred minimum value, whereas an upper bound is the preferred maximum value provided by the decision-maker for the corresponding objective. Accordingly, we proposed a novel interactive method incorporating the preferred ranges in the solution process. Then, we generated different compromise solutions reflecting these decision-makers’ preferences as well as possible. In this step, we introduced two kinds of solutions, considering that finding a solution that meets all the decision-maker’s preferences may not be possible. The first kind of solution (group I) meets all the desired preferred ranges, whereas the second kind of solution (group II) only meets some preferred ranges. Even though solutions in the latter group violate some preferred ranges, the decision-maker gets more insight into the trade-offs in the compromise solutions. In this way, the decision-maker can learn what is achievable and what is not. Different visualizations have been utilized to illustrate solutions in the multiobjective optimization literature [28,29]. In this paper, we visualized the solutions with parallel coordinate plots, representing several objectives and solutions simultaneously [30].

In different iterations, the decision-maker can update one’s preferences based on the increasing understanding of the available therapies and the existing trade-offs between the objectives. The solution process continues until the decision-maker is satisfied and has found the most preferred therapy.

## Results

**Figure 3.**
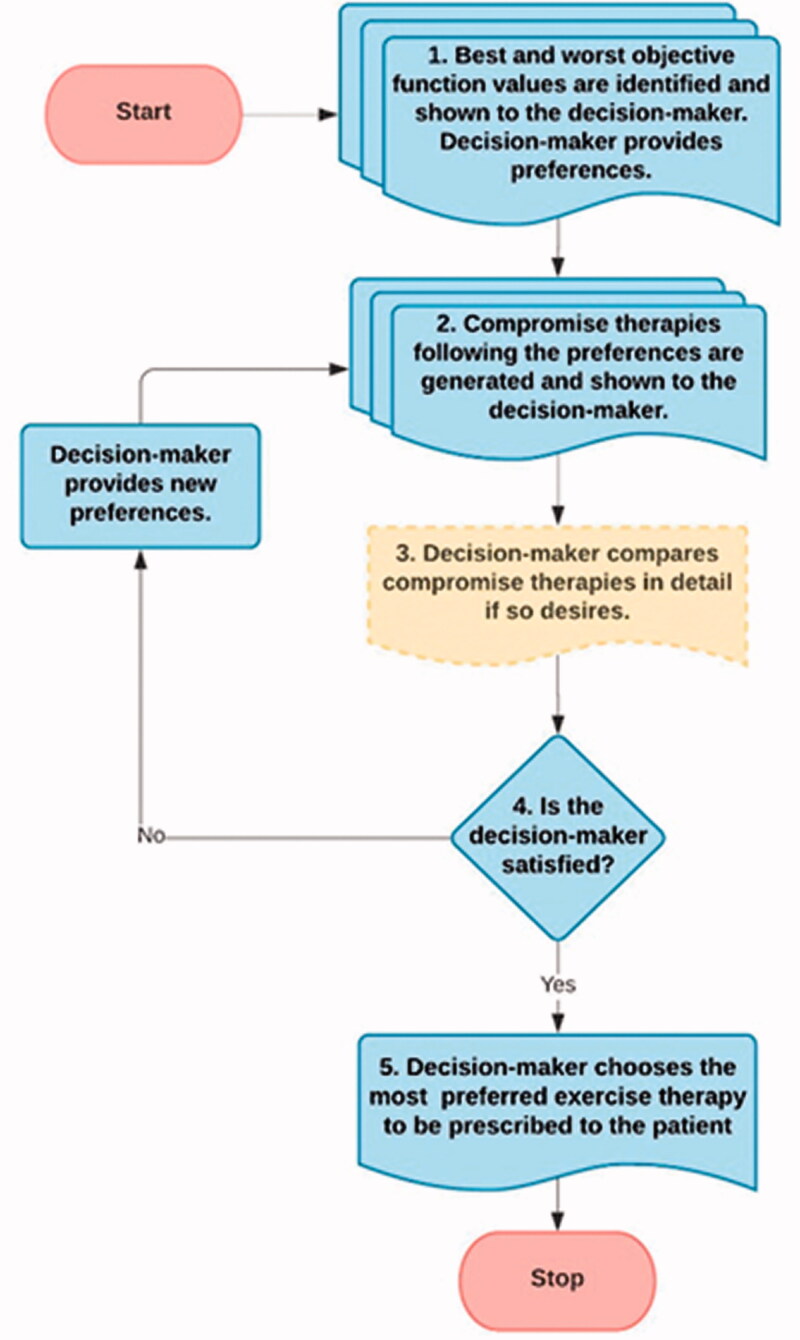
Overview of the interactive method.

This section demonstrates the proposed interactive method and how it can be used as a decision support tool in finding the most suitable compromise therapy for the problem formulated. Figure 3 depicts the iterative steps and some other steps of the interactive method to support decision-making in knee OA. The steps are described below. The technical details of the proposed interactive method are given in Supplementary Material C.

**Step 1.** The best and the worst values of each objective are identified and shown to the decision-maker to give an overview of the range of feasible values. Then, the decision-maker provides his/her preference information as a preferred range for each objective.
**Step 2.** The multiobjective optimization problem is solved, and the desired number of compromise therapies (reflecting the preferences as well as possible) is shown to the decision-maker. In the visualization, group I solutions are highlighted, whereas the others (group II) are represented in shading, meaning that some sacrificing in some preferred ranges are needed to obtain the desired values offered by these solutions in some other objectives. Note that if the objective values are better than the decision-maker desired values, the relevant solutions are still counted as the group I solutions.
**Step 3 (optional).** The decision-maker can compare and analyze the compromise exercise therapy modalities in more detail, such as by checking the exercises from the clinical aspects, if so desired.
**Step 4.** If the decision-maker wants to continue and provide different preferences, the solution process continues from *Step 2*. Alternatively, if the decision-maker is satisfied with the current compromise exercise therapy modalities, the process continues from *Step 5*.
**Step 5.** Finally, after analyzing the compromise therapies, the decision-maker prescribes the most preferred and suitable exercise therapy modality according to the patient’s needs and clinical status. This ends the solution process.

The source code (using Python as the programming language running in Jupyter notebook^1^ is openly accessible at the GitHub repository.^2^ The data used in the solution process are available in Table 1 in Supplementary Material A. In the beginning, the decision-maker was informed of the terminology used, the idea of the interactive solution process, and how one can provide preference information, as presented in Figure 3. As our subject, we considered a patient with mild knee OA who had pain in his knees and some physical difficulty in the daily routines. His budget was around 300€. However, he could bear up to 600€ if his physical functionality and pain could be improved by at least 25% in two/three months. In addition, he preferred to have as few supervised sessions as possible because of the distance, although he would participate in as many sessions as needed. Moreover, his current health status and clinical background did not show any severe disease or limitations in performing high-performance exercises. Note that at this stage, based on the patient characteristics and recent clinical guidelines, the decision-maker could set what types of exercises (e.g. resistance, aerobic, stretching, water-based, etc.) should be considered and/or excluded from the consideration. However, in this experiment, the decision-maker preferred to consider all available exercises. Given this information, the decision-maker was asked to provide a preferred range for each objective at each iteration of the solution process. As mentioned, these preferences are incorporated in solving the multiobjective optimization problem to find multiple compromise therapies that reflect the preferences of the decision-maker that are set based on the patient’s characteristics.

The solution process was started by showing the best and worst values of each objective to the decision-maker to inform him of the ranges of the available therapies. Then, the decision-maker provided the number of solutions (four solutions) to be shown at each iteration and the preferred ranges for each objective. First, the decision-maker set the cost between 300€ and 600€ based on the patient budget. The decision-maker wished to find therapies that improve pain reduction and functionality by 30 and 25%, respectively. For both of these objectives, the minimum improvement was set to 15%. According to his previous experiences and the patient’s requests, he prefers self-exercises that could be quickly performed. Therefore, the desired values of the fourth and fifth objectives were given near to their minimum observed values (0 and 8, respectively). The upper bounds for these objectives are specified as 15 supervised sessions and 26 weeks.

**Figure 4.**
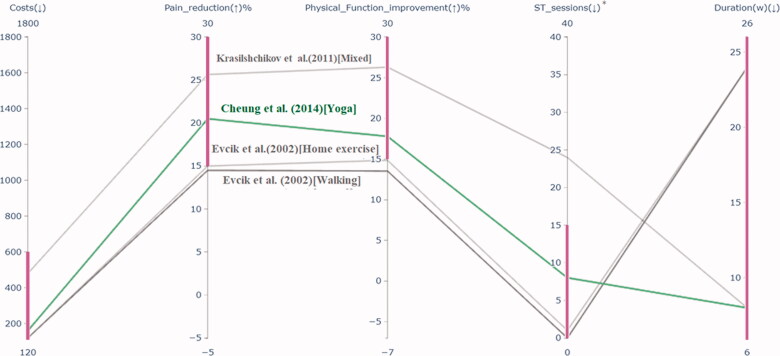
Therapies reflecting the given preference information in the 1st iteration. *ST_sessions: number of supervised training sessions.

After the first iteration, only one solution met all the desired ranges (group I) and is highlighted in Figure 4. The decision-maker wanted to see four solutions. Thus, three more therapies (from group II) were found and shown in the figure in shading to make the decision-maker aware of other possible solutions near his preferred ranges.

The decision-maker could easily compare the solutions, given that their number is fairly low. Because all solutions shown are compromise therapies, the decision-maker was aware that something has to be sacrificed to improve some other objectives. The therapy best matched the preferences (a yoga type exercise described by Cheung et al. [31]) was not satisfactory for the decision-maker, given that other solutions showed better improvement in pain reduction and function, although they had more supervised training sessions. An example is a therapy proposed by Krasilshchikov et al. [32], as shown in Figure 4.

After having seen the solutions of the first iteration, the decision-maker was interested in a better functionality improvement than pain reduction. The decision-maker was willing to sacrifice the number of supervised sessions and duration of the treatment for getting such therapies. Therefore, he provided new preference information for the objectives, as shown in Table 2.

**Table 2.** Preferences in different iterations.

		Costs (€)	Pain change (%)	Function change (%)	Supervised sessions	Period (weeks)
Iteration 1	Preferred ranges	300	+30%	+25%	0	8
		600	+15%	+15%	15	26
Iteration 2	Preferred ranges	200	+25%	+40%	0	12
		500	+15%	+15%	30	26

**Figure 5.**
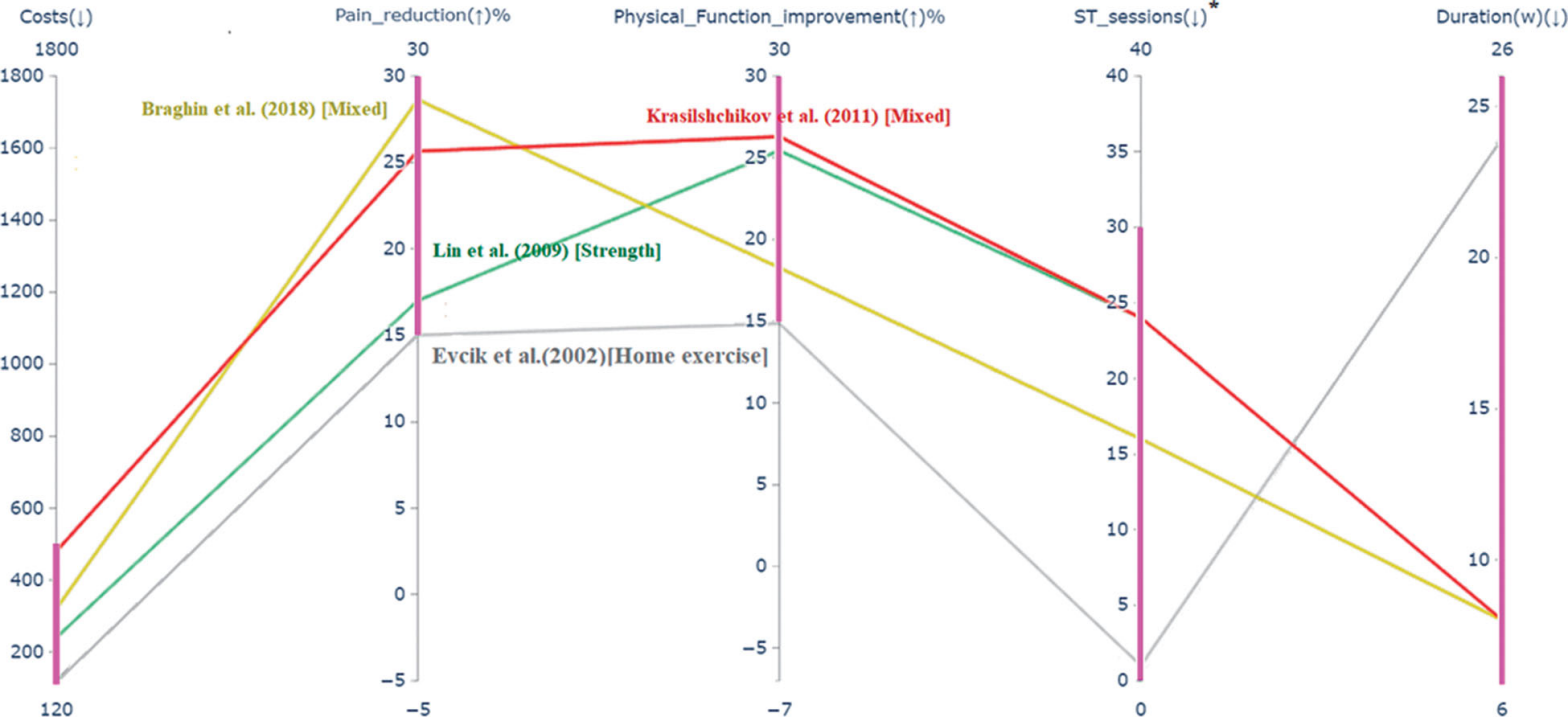
Therapies reflecting the given preference information in the 2nd iteration. *ST_sessions: number of supervised training sessions.

At the second iteration, three group I solutions reflected the preferences well, which were described by Krasilshchikov et al. [32], Braghin et al. [33], and Lin et al. [34], were obtained and are highlighted in Figure 5. Given that the decision-maker wanted to see four solutions, one more solution [35] (from group II) was found closest to the provided preference information. The decision-maker selected the combined resistance and aerobic exercise program described by Krasilshchikov et al. [32] as the most preferred solution from these four compromise therapies. It had the most significant functionality improvement and also improved pain reduction effectively. However, to achieve these improvements, one needs to pay more money and take more supervised sessions than other solutions. Thus, based on clinical outcomes and patient characteristics, in this case, the personalized choice was a mixed type of exercise therapy modality described by Krasilshchikov et al. [32]. Comparing a subset of solutions (3–5) in our interactive approach helped the decision-maker in assessing the objectives simultaneously. He reached a satisfactory solution in only two iterations.

## Discussion

This study proposes a decision support tool utilizing multiobjective optimization to help decision-makers identify the most appropriate exercise therapy modality for patients with knee OA. The goodness of therapies is characterized by considering multiple conflicting objectives simultaneously. Hence, e.g. both best cost and best effect are to be sought simultaneously. The domain expertise of a decision-maker augmented characteristics of the desirability of therapies. Although the decision-maker could set different objectives to be considered in the proposed decision support tool, based on the data availability and the decision-maker wishes in our case, we considered five objectives in selecting exercise therapy modalities: cost of individual therapies, WOMAC pain and function, number of supervised exercise sessions, and length of treatment period were the conflicting objectives. As mentioned earlier, although supervised exercise therapy modalities are recommended, mainly to prevent exercise adherence dropout, various challenges exist in planning supervised sessions. For instance, some patients (e.g. because of disability, additional time and expenses, travel distances, quarantine limitations caused by an epidemic or pandemic, such as Coronavirus disease 2019) or physiotherapists’ lack of resources at work often prefer to have a few (physical) supervised sessions as possible. For these reasons and to pay particular attention to this objective, we separated the fourth and the fifth objectives.

Moreover, one should note that this study should be considered as a methodological development and proof of concept because we need to develop novel methods to promote personalized recommendations based on the latest theoretical and technological developments. Accordingly, the objectives, exercises, and any other parameters considered in this study could also be selected differently if some other aspects characterize the efficacy of therapies better. For example, some more objectives, such as improvement in performance and QoL, or some other exercises can be added to the proposed decision support tool if desired, and the relevant data are available. As mentioned, our selection of objectives is explained by the data available.

As mentioned earlier, because of the conflicting nature of the objectives, instead of a single optimal solution, multiobjective optimization problems have several compromise solutions with different trade-offs. In this case, solutions refer to therapies. Therefore, decision-makers can find suitable compromises with the help of a multiobjective optimization method and finally choose the most preferred exercise therapy modality among the compromises found based on his/her analysis and patient characteristics. Naturally, the preferences of decision-makers are specific to each patient. Thus, the choice depends on different symptoms and needs (e.g. a patient with chronic pain but rather good functionality may need a different treatment from another who has less pain but needs more functionality). Based on the preferences, in each case, various compromise solutions will be found and presented to the decision-maker, and the final choice will be different accordingly. Therefore, the patient characteristics in question affect the preferences of the decision-maker and the most preferred therapy as the final choice. Indeed, what we described in the results section was only a proof of concept to illustrate how the proposed methodology can be utilized.

To the best of our knowledge, this study is the first to use multiobjective optimization to support decision-making in selecting exercise therapy modalities for patients with knee OA. This novelty can be counted as the new wave of digitalization and data analytics that connect researchers from different disciplines to make the best use of data and improve traditional methods to select intervention types that should be most beneficial and cost-effective for each patient. Therefore, we cannot compare our results with previous studies using multiobjective optimization or similar optimization methods in this patient group. The results of this study are not comparable with the traditional meta-analyses in the treatment of knee OA patients, as they have been conducted on several clinical trials in an effort to obtain higher statistical power with more substantial evidence on the possible effectiveness for the outcomes than from any individual study.

Multiobjective optimization, in turn, offers one or more individual study solutions based on the objectives that have been given for the desired therapy objectives. Ideally, the individual studies obtained from the multiobjective optimization solution process should be in line with the recommendations and guidelines for the management of knee OA. For example, the yoga exercise [31] found in the first iteration is in agreement with the current treatment guidelines that land-based exercises and mind–body exercises, such as tai chi and yoga, are effective and safe for all patients with knee OA [36]. Similarly, the other suggested study by Krasilshchikov et al. [32] found in the second iteration is also concordant with the results of some recent meta-analyses showing that aerobic exercise in combination with strengthening exercises is efficient in pain reduction and function improvement [37]. However, note that the studies by Cheung et al. [31] and Krasilshchikov et al. [32] had small sample sizes. In addition, the lack of individual data prevented us from considering net changes in variances, as they are not linear operations, and the covariance information was not available. Therefore, in the current version of the proposed decision support tool, the decision-maker should conduct these kinds of analyses before or after the optimization process. The decision-makers can also explore various possibilities and use their expertise to find a meaningful modality best suited for each patient based on their needs and characteristics, thanks to the interactive nature of the proposed tool. Naturally, as always in data-driven decision support, the goodness of the performance of the decision support tools depends on the quality of the data available.

As stated, our aim in this paper was not to find a general recommendation of the best (type of) exercise modalities but to develop a methodology (and corresponding prototype of a decision support tool) leading to more personalized recommendations based on patients’ needs and characteristics. Therefore, the proposed decision support tool first compares and evaluates the goodness of the available modalities based on their outcome values on the (five) selected objectives and the decision-maker’s preferences reflecting the patient’s needs. Then, the tool recommends a few alternative modalities that follow the preferences (individual patient’s needs and characteristics) best. Thus, the decision-maker must make the final decision and pick the best-fitted one based on their expertise, the patient’s needs, and the latest clinical guidelines (e.g. OARSI/ESCEO/EULAR).

## Strengths and limitations

The strength of the proposed decision support tool is the ability to make better decisions by considering conflicting objectives simultaneously. On the one hand, the fact that we used trial-specific mean results can be considered a strength for generalizability. On the other hand, it is a limitation because of two reasons: (i) clinical heterogeneity between the trials may exist, such as different mean OA stages, influencing trial outcomes; and (ii) we may not take into account all clinically relevant individual data.

A further limitation arises from the characteristics of the data available. The data derived from the published papers are means and standard deviations of each therapy’s outcomes. This limits the proposed decision support tool, as it cannot provide personalized recommendations considering all relevant patient characteristics. Specifically, we could not consider more objectives (such as QoL and net changes in the variances) and/or other essential characteristics, such as comorbidities (e.g. chronic pain and hypertension), because of the lack of data. However, the proposed decision support tool can be adjusted for different objectives, exercise therapy modalities, and patients or even adapted for various diseases. Therefore, if we had relevant individual data from existing studies, we could consider such information in our proposed decision support tool to improve its reliability and help decision-makers make better decisions in practice, considering all the essential patients’ characteristics and health status in more detail.

In this paper, we tested the proposed methodology with only one decision-maker. However, the proposed methodology and the decision support tool can be extended to be used in group decision-making if augmented with an extra step of utilizing a suitable technique, such as Nominal group technique [38,39] or Delphi survey [40,41] (Also see [42,43] for more information about multiobjective group decision-making techniques).

Besides, we utilized all the available exercise modalities without any pre-evaluation and the results are subjective to the data and the solitary case trial. In practice, the clinicians should do primary filtering of (types of) modalities before utilizing the tool following the latest clinical guidelines and meta-analyses. Since the tool itself does not perform such an analysis (assuming this step precedes applying the tool), it is still the decision-maker's responsibility to consider the latest clinical guidelines before the final decision.

## Conclusions and future directions

A new decision-support tool for determining the most preferred exercise therapy modality for knee OA was proposed. As a part of it, a novel interactive multiobjective optimization method was introduced. Even though the focus was on the field of rehabilitation medicine, the proposed methodology can be utilized in any field of medical and healthcare services, where several alternative treatment options for specific conditions and data about them exist. Our intention is not to provide any global answer or recommendation but a decision support tool. An interested decision-maker can use the proposed decision support tool and analyze the suggested therapies (by the tool) by incorporating one’s preferences (reflecting patient’s needs and health status) and prescribe the most appropriate one to the patient in question. We demonstrated and tested the proposed decision support tool to demonstrate its benefits and usability in prescribing exercise therapy modalities applied to treating knee OA patients. However, the proposed tool was tested with a limited dataset extracted from a systematic review of different trials (trial Mean effects used), for a methodology feasibility check as a proof of concept. Thus, further validation with more interventions, individualized data, and various decision-makers are required before any practical utilization.

Using interactive multiobjective optimization methods in their current form requires an analyst in addition to a domain expert to pre-process the data and formulate the optimization problem. A further step in the development could be creating a user-friendly interface, which does not necessitate the presence of an analyst. Moreover, if we had individual data of each participant in the considered RCTs, the optimization process could be performed in a more personalized way. This approach would improve the accuracy of the model and save the time of the decision-maker in the final analysis.

Besides, clinicians need to be more open to new methods and make the data openly and anonymously available to make advanced tools, such as artificial intelligence and machine learning techniques, applicable. Unfortunately, almost all available machine learning techniques require a large number of samples (e.g. hundreds to thousands) for initial training to be able to provide an accurate prediction. So, they cannot be directly applied in exercise training that often has been conducted with a few tens of patients. More developments in machine learning are also needed to handle small datasets like cases in exercise RCTs. However, before that, the restrictions in clinical data must be relaxed. In this regard, anonymous data can be the way.

In summary, designing a user-friendly interface, considering more objectives (like performance and QoL), and using individualized patient data are our future research directions. Furthermore, combining the proposed methodology with machine learning techniques and extending it in comparing the efficacy of any other treatment in various diseases is another promising future direction of this research.

## Supplementary Material

Supplemental Material

## Data Availability

The data used in this study are openly available in the supplementary materials. The source code is openly accessible at the GitHub repository: https://github.com/industrial-optimisation-group/Interactive-Multiobjective-Optimisation-for-Finding-the-Most-Suitable-Exercise-Therapy-in-Knee-Osteo.

## Notes

1
https://jupyter.org

2
https://github.com/industrial-optimization-group/Interactive-Multiobjective-Optimization-for-Finding-the-Most-Suitable-Exercise-Therapy-in-Knee-Osteo
