# Supplementary material for: Interactive multiobjective optimization for finding the most preferred exercise therapy modality in knee osteoarthritis
Source: Ann Med. 2022 Jan 13;54(1):181–94. doi: 10.1080/07853890.2021.2024876 (PMC8759734; doi:10.1080/07853890.2021.2024876)
Supplement: Supplemental Material [file IANN_A_2024876_SM1855.zip › Supplementary_material_A_B_and_C_knee_OA_22.6.2020_.pdf]

Interactive Multiobjective Optimization for Finding the Most Suitable Exercise Therapy in Knee Osteoarthritis Supplementary Material A-C

Babooshka Shavazipour[1], Bekir Afsar[1], Juhani Multanen[2,3], Urho Kujala[2], and Kaisa Miettinen[1]

[1] *University of Jyvaskyla, Faculty of Information Technology, P.O. BOX 35 (Agora), FI-40014 University of Jyvaskyla, Finland*
[2] *University of Jyväskylä, Faculty of Sport and Health Sciences, P.O. BOX 35, FI-40014 University of Jyvaskyla, Finland*
[3] *Central Finland Central Hospital, Department of Physical Medicine and Rehabilitation, Keskussairaalantie 19, FI-40620 Jyvaskyla, Finland*

A Selected papers

	Authors	Therapy	Cost	Pain change	Function. change	Supervised sessions	Periods (w)
1	An et al. [1]	Baduanjin	400.0	4.024000	11.172	40	8
2	Bennell et al. [2]	Home exercise	300.0	2.400	6.8000	7	12
3	Braghin et al. [3]	Mixed	320.0	5.728	12.4236	16	8
4	Cheung et al. [4]	Yoga	160.0	4.100	12.1000	8	8
5	Jorge et al. [5]	Resistance	480.0	4.300000	8.700	24	12
6	Jorge et al. [5]	Resistance	240.0	3.100	3.3000	12	6
7	Topp et al. [6]	Resistance	240.0	1.390000	2.990	16	16
8	Topp et al. [6]	Resistance	240.0	1.710000	6.620	16	16
9	Fransen et al. [7]	Individual training	350.0	1.840	1.3400	7	8
10	Fransen et al. [7]	Group (Mixed)	320.0	2.300000	1.720	16	8
11	Wallis et al. [8]	Walking	170.0	0.900000	-1.000	13	12
12	Salacinski et al. [9]	Cycling	440.0	2.980000	2.540	24	12
13	Wortley et al. [10]	Resistance	400.0	2.840000	6.080	20	10
14	Wortley et al. [10]	Tai JI	400.0	0.600000	2.800	20	10
15	O'Reilly et al. [11]	Mixed	150.0	1.870000	3.560	0	26
16	Simão et al. [12]	Platform group	180.0	5.500	9.9200	36	12
17	Simão et al. [12]	Squat group	180.0	5.000000	3.100	36	12
18	Lee et al. [13]	Tai Chi Qigong	320.0	1.142857	5.440	16	8
19	Lin et al. [14]	Proprioceptive	480.0	2.500000	8.600	24	8
20	Lin et al. [14]	Strength	240.0	3.400	17.3000	24	8
21	Aglamış et al. [15]	Mixed	720.0	-1.00000	-1.000	36	12
22	Lim et al. [16]	Strength-Malaligned	525.0	0.320000	0.100	7	12
23	Lim et al. [16]	Strength-Neutrally aligned	525.0	2.380000	5.400	7	12
24	Lee and Lee [17]	Tai Chi Sun-style	480.0	3.700000	-4.800	24	12
25	de Rooij et al. [18]	Mixed	300.0	1.400000	6.600	20	10
26	de Rooij et al. [18]	Mixed	600.0	2.600000	9.200	40	20
27	Jan et al. [19]	High-resistance	1200	2.500000	8.800	24	8
28	Jan et al. [19]	Low-resistance	1800	1.800000	8.400	24	8
29	Evcik and Sonel [20]	Home exercise	120.0	3.000	10.1000	1	24
30	Evcik and Sonel [20]	Walking	120.0	2.900	9.2000	0	24
31	Krasilshchikov et al. [21]	Mixed	480.0	5.130	17.8800	24	8

Table 1: Selected papers satisfying the inclusion criteria

B Mathematical formulation of the multiobjective optimization problem

The proposed multiobjective optimization problem can be formulated as follows:

$$
\begin{array}{ll}
\text{minimize} & f_1(\mathbf{x}) = \sum_{i=1}^{n} C_i x_i \\
\text{maximize} & f_2(\mathbf{x}) = \sum_{i=1}^{n} [\mathbf{E}(P_i^{Pre(EG)} - P_i^{Post(EG)}) - \mathbf{E}(P_i^{Pre(CG)} - P_i^{Post(CG)})] x_i \\
\text{maximize} & f_3(\mathbf{x}) = \sum_{i=1}^{n} [\mathbf{E}((Ph)_i^{Pre(EG)} - (Ph)_i^{Post(EG)}) - \mathbf{E}((Ph)_i^{Pre(CG)} - (Ph)_i^{Post(CG)})] x_i \\
\text{minimize} & f_4(\mathbf{x}) = \sum_{i=1}^{n} ST_i x_i \\
\text{minimize} & f_5(\mathbf{x}) = \sum_{i=1}^{n} T_i x_i \\
& \\
\text{s.t.} & \\
& \sum_{i=1}^{n} x_i = 1 \\
& x_i \in \{0, 1\}, \quad i = 1, \dots, n,
\end{array} \tag{1}
$$

where $C_i, i = 1, \dots, n$, is the cost of the ith therapy x_i (we have n therapies), $\mathbf{E}(P_i^{Pre(EG)} - P_i^{Post(EG)})$ is the expected value of the differences between the mean of WOMAC pain scores pre- and post-intervention. Here, $\mathbf{E}(.)$ refers to the expected value. 'EG' and 'CG' describe the exercise and control groups, respectively. Furthermore, $\mathbf{E}((Ph)_i^{Pre(EG)} - (Ph)_i^{Post(EG)})$ is the expected value of the change/improvement in WOMAC physical functionality scores after the exercise period. Moreover, $ST_i, i = 1, \dots, n$, is the number of supervised training sessions of ith therapy and $T_i, i = 1, \dots, n$ is the length of the ith therapy (in weeks). Finally, $\mathbf{x} = x_1, \dots, x_n$, is the vector of n decision variables referring different types of exercise therapy and

$$
x_i = \begin{cases} 1, & \text{if relevant therapy is selected} \\ 0, & \text{otherwise.} \end{cases}
$$

The constraint in the problem makes sure that only one therapy is selected.

C Solution method

Over the years, many interactive methods have been developed in the literature to solve multiobjective optimization problems. An example of differences between methods is in the different type of preference information utilized. In this study, on one hand, the DM preferred to provide preferred ranges (upper and lower bounds) for each objective function. On the other hand, he favored seeing multiple solutions (therapies) in each iteration of the interactive solution process. Accordingly, we propose a novel interactive method that supports both these DM's wishes. For the latter request, the proposed method is inspired by the NIMBUS method [22]. However, for the former demand, a different type of preference information must be considered.

Similar to many multiobjective optimization methods [23, 24], the main idea is to convert the multiobjective optimization problem into a single objective one (also called a scalarized problem). However, to generate multiple solutions in each iteration of the proposed interactive method, like the NIMBUS method, several scalarization functions are utilized. As mentioned in [25], numerous scalarization functions have been developed in the literature. In this paper, the reference point-based scalarization functions are considered, in which, the DM determines desirable values for each objective function. Since the available preference information, in our study, is the preferred ranges, we replace the desirable objective values (also called aspiration levels) utilized in reference point-based scalarization functions by desirable upper/lower bounds

(depending on the minimization/maximization form of the objective functions). Therefore, by using any reference point-based scalarization functions, the relevant scalarized multiobjective optimization problem can be solved utilizing broadly developed single objective methodologies [26].

As also described in the main text, besides the aspiration levels, the other side of the desired ranges, which represents the objective values the DM would like to evade (know as reservation levels in the multiobjective optimization literature), are also considered in the proposed method.

To increase the chance of obtaining different solutions, we utilize the results of [25], which is also followed by the NIMBUS method. Miettinen and Mäkelä [25] compared fifteen different scalarization functions from both theoretical and numerical perspectives. Accordingly, we chose an achievement scalarization function (ASF) [27, 28], and scalarizing functions from the satisficing trade-off method (STOM) [29, 30], the GUESS [31], and the Step method (STEM) [32]. (The first three are utilized by the NIMBUS method as well.) One should note that, here, by the names (e.g., STOM) we refer to the scalarization functions and not the methods these scalarization functions originate from.

We consider four different variants of ASFs to enhance the possibility of producing more diverse solutions, still reflecting preferences. The differences between some variants of ASF is only in their normalization scheme. We note that the normalization/weighting schemes are only designed to avoid bias effects that may be caused by various scales of objective functions and no preference information, of any kind, is involved [33]. The most common variant which is considered called range normalization [33, 34]. As the second variant, we use the ideal normalization scheme [33, 35] while no normalization scheme is considered in the third variant. Finally, to directly consider both upper and lower bounds of the preferred ranges within the solution generation process, the variant of ASF utilizing both aspirations and reservations levels (originally proposed by Wierzbicki [36], Wierzbicki et al. [37], and also used in the PIE method [38]) is usec as the fourth variant of ASF. Note that, considering these seven different scalarization functions are not guaranteed to generate seven different solutions as, depending on the problem, some of them may end up with the same solution (to see how different scalarization functions generate different solutions, we refer the reader to [25, 33]). Therefore, in each iteration of the proposed interactive method, we can produce up to seven different solutions (usually fewer), incorporating the DM's preferred ranges. We generate and show as many solutions as the DM desires.

References

[1] An B, Dai K, Zhu Z, Wang Y, Hao Y, Tang T, et al. Baduanjin alleviates the symptoms of knee osteoarthritis. The Journal of Alternative and Complementary Medicine, 14(2):167–174, 2008.

[2] Bennell KL, Hunt MA, Wrigley TV, Hunter DJ, McManus FJ, Hodges PW, et al. Hip strengthening reduces symptoms but not knee load in people with medial knee osteoarthritis and varus malalignment: a randomised controlled trial. Osteoarthritis and Cartilage, 18(5):621–628, 2010.

[3] Braghin RMB, Libardi EC, Junqueira C, Nogueira-Barbosa MH, and de Abreu DCC. Exercise on balance and function for knee osteoarthritis: a randomized controlled trial. Journal of Bodywork and Movement Therapies, 22(1):76–82, 2018.

[4] Cheung C, Wyman JF, Resnick B, and Savik K. Yoga for managing knee osteoarthritis in older women: a pilot randomized controlled trial. BMC Complementary and Alternative Medicine, 14(1):160, 2014.

[5] Jorge RTB, de Souza MC, Chiari A, Jones A, Fernandes ARC, Júnior IL, et al. Progressive resistance exercise in women with osteoarthritis of the knee: a randomized controlled trial. Clinical Rehabilitation, 29(3):234–243, 2015.

[6] Topp R, Woolley S, Hornyak III J, Khuder S, and Kahaleh B. The effect of dynamic versus isometric resistance training on pain and functioning among adults with osteoarthritis of the knee. Archives of Physical Medicine and Rehabilitation, 83(9):1187–1195, 2002.

[7] Fransen M, Crosbie J, and Edmonds J. Physical therapy is effective for patients with osteoarthritis of the knee: a randomized controlled clinical trial. The Journal of Rheumatology, 28(1):156–164, 2001.

[8] Wallis JA, Webster KE, Levinger P, Singh PJ, Fong C, and Taylor NF. A walking program for people with severe knee osteoarthritis did not reduce pain but may have benefits for cardiovascular health: a phase II randomised controlled trial. Osteoarthritis and Cartilage, 25(12):1969–1979, 2017.

[9] Salacinski AJ, Krohn K, Lewis SF, Holland ML, Ireland K, and Marchetti G. The effects of group cycling on gait and pain-related disability in individuals with mild-to-moderate knee osteoarthritis: a randomized controlled trial. Journal of Orthopaedic & Sports Physical Therapy, 42(12):985–995, 2012.

[10] Wortley M, Zhang S, Paquette M, Byrd E, Baumgartner L, Klipple G, et al. Effects of resistance and Tai Ji training on mobility and symptoms in knee osteoarthritis patients. Journal of Sport and Health Science, 2(4):209–214, 2013.

[11] O'Reilly SC, Muir KR, and Doherty M. Effectiveness of home exercise on pain and disability from osteoarthritis of the knee: a randomised controlled trial. Annals of the rheumatic diseases, 58(1):15–19, 1999.

[12] Simão AP, Avelar NC, Tossige-Gomes R, Neves CD, Mendonça VA, Miranda AS, et al. Functional performance and inflammatory cytokines after squat exercises and whole-body vibration in elderly individuals with knee osteoarthritis. Archives of Physical Medicine and Rehabilitation, 93(10):1692–1700, 2012.

[13] Lee HJ, Park HJ, Chae Y, Kim SY, Kim SN, Kim ST, et al. Tai Chi Qigong for the quality of life of patients with knee osteoarthritis: a pilot, randomized, waiting list controlled trial. Clinical Rehabilitation, 23(6):504–511, 2009.

[14] Lin DH, Lin CHJ, Lin YF, and Jan MH. Efficacy of 2 non-weight-bearing interventions, proprioception training versus strength training, for patients with knee osteoarthritis: a randomized clinical trial. Journal of Orthopaedic & Sports Physical Therapy, 39(6):450–457, 2009.

[15] Lim BW, Hinman RS, Wrigley TV, Sharma L, and L Bennell K. Does knee malalignment mediate the effects of quadriceps strengthening on knee adduction moment, pain, and function in medial knee osteoarthritis? a randomized controlled trial. Arthritis Care & Research: Official Journal of the American College of Rheumatology, 59(7):943–951, 2008.

[16] Aglamış B, Toraman NF, and Yaman H. The effect of a 12-week supervised multicomponent exercise program on knee OA in Turkish women Journal of Back and Musculoskeletal Rehabilitation, 21(2):121–128, 2008.

[17] Lee HY and Lee KJ. Effects of tai chi exercise in elderly with knee osteoarthritis. Journal of Korean Academy of Nursing, 38(1):11–18, 2008.

[18] de Rooij M, van der Leeden M, Cheung J, van der Esch M, Häkki-nen A, Haverkamp D, et al. Efficacy of tailored exercise therapy on physical functioning in patients with knee osteoarthritis and comorbidity: a randomized controlled trial. Arthritis Care & Research, 69(6):807–816, 2017.

[19] Jan MH, Lin JJ, Liau JJ, Lin YF, and Lin DH. Investigation of clinical effects of high-and low-resistance training for

patients with knee osteoarthritis: a randomized controlled trial. Physical Therapy, 88(4):427–436, 2008.

[20] Evcik D and Sonel B. Effectiveness of a home-based exercise therapy and walking program on osteoarthritis of the knee. Rheumatology International, 22(3):103–106, 2002.

[21] Krasilshchikov O, Shaw I, Sungkit NB, Shaw BS, and Shihabudin TM. Effects of an eight-week training programme on pain relief and physical condition of overweight and obese women with early stage primary knee osteoarthritis: physical activity, health and wellness. African Journal for Physical Health Education, Recreation and Dance, 17(2):328–339, 2011.

[22] Miettinen K and Mäkelä MM. Synchronous approach in interactive multiobjective optimization. European Journal of Operational Research, 170(3):909–922, 2006.

[23] Chankong V and Haimes YY. Multiobjective decision making: theory and methodology. Publishing Co., Inc., New York, 1983.

[24] Miettinen K. Nonlinear Multiobjective Optimization. Kluwer Academic Publishers, 1999.

[25] Miettinen K and Mäkelä MM. On scalarizing functions in multiobjective optimization. OR Spectrum, 24(2):193–213, 2002.

[26] Bazaraa MS, Sherali HD, and Shetty CM. Nonlinear programming: theory and algorithms. John Wiley & Sons, Hoboken, New Jersey, third editio, 2013.

[27] Wierzbicki AP. The use of reference objectives in multiobjective optimization. In Günter Fandel and Tomas Gal, editors, Multiple criteria decision making theory and application, pages 468–486. Springer, Berlin Heidelberg New York, 1980.

[28] Wierzbicki AP. A mathematical basis for satisficing decision making. Mathematical modelling, 3(5):391–405, 1982.

[29] Nakayama H. Aspiration level approach to interactive multi-objective programming and its applications. In PM Pardalos, Y Siskos, and Zopounidis C, editors, Advances in Multicriteria Analysis, pages 147–174. Kluwer, 1995.

[30] Nakayama H and Sawaragi Y. Satisficing trade-off method for multiobjective programming. In Grauer M and Wierzbicki AP, editors, Interactive Decision Analysis, pages 113–122. Springer, Berlin Heidelberg New York, 1984.

[31] Buchanan JT. A naive approach for solving mcdm problems: The GUESS method. Journal of the Operational Research Society, 48(2):202–206, 1997.

[32] Benayoun R, De Montgolfier J, Tergny J, and Laritchev O. Linear programming with multiple objective functions: Step method (STEM). Mathematical programming, 1(1):366–375, 1971.

[33] Ruiz F, Luque M, and Cabello JM. A classification of the weighting schemes in reference point procedures for multiobjective programming. Journal of the Operational Research Society, 60(4):544–553, 2009.

[34] Steuer RE. Multiple Criteria Optimization: Theory, Computation, and Application. John Wiley and Sons, 1986.

[35] Osyczka A. Multicriterion optimization in engineering with FORTRAN programs. Ellis Harward Lim. Publ., Chichester, 1984.

[36] Wierzbicki AP. On the completeness and constructiveness of parametric characterizations to vector optimization problems. OR Spektrum, 8(2):73–87, 1986.

[37] Wierzbicki AP, Makowski M, and Wessels J. Model-based decision support methodology with environmental applications, 2000.

[38] Sindhya K, Ruiz AB, and Miettinen K. A preference based interactive evolutionary algorithm for multi-objective optimization: PIE. In H.C. Takahashi, K Deb, E.F. Wanner, and S Greco, editors, Evolutionary Multi-Criterion Optimization: 6th International Conference, pages 212–225, Berlin, Heidelberg, 2011. Proceedings, Springer-Verlag.
