# Supplementary material for: Interactive multiobjective optimization for finding the most preferred exercise therapy modality in knee osteoarthritis
Source: Ann Med. 2022 Jan 13;54(1):181–94. doi: 10.1080/07853890.2021.2024876 (PMC8759734; doi:10.1080/07853890.2021.2024876)
Supplement: Supplemental Material [file IANN_A_2024876_SM1855.zip › Supplementary_material_D_cost_assessment.pdf]

Supplementary material D: Cost-Evaluation of Exercise Therapies for Patients with Knee Osteoarthritis

Cost analysis is based on individual, i.e. patient elements of the price

Cost is measured from the time between baseline and end-point of the outcome measure (follow-ups are not included)

ESTIMATE OF THE INDIVIDUAL COST IS BASED ON THE FOLLOWING:

- Fee for physiotherapy (or similar exercise instructor);
    - individual 1 hour 100 €
    - individual 45 min 75 €
    - individual 30 min 50 €
    - individual (group 1/5) 1 hour 20 €
    - individual (group 1/5) 45 min 15 €
    - individual (group 1/5) 30 min 10 €

- Non-supervised training: rehabilitation center, gym, etc;
    - individual /session 10 €

- Non-supervised training: home
    - individual / session 0 €

- Phone call /SMS message etc. 10 €

- Exercise equipments; 0 €

Calculations

An et al. (2008)[1]

Exercise group: Number of sessions 40 under supervision of physiotherapist, length of session 30 min, number of trainees in the exercise group, n = 14:

Control group: no intervention, n=14

- ➔ EG: 40 sessions * 10 € /group individual = 400 €
- ➔ CG: no costs

Bennell et al. (2010)[2]

Exercise group: Number of unsupervised sessions at home 60, length of session 30 min, number of individually supervised sessions 7, length of session 15 min, number of trainees in the exercise group, n = 45:

Control group: No intervention, n=44

- ➔ EG: home exercise 0 €
- ➔ EG: (5 sessions * 50 € + 2 sessions * 25/ individ.) = 300 €
- ➔ CG: no costs

Braghin et al. (2018)[3]

Exercise group I (symptomatic): supervised 60 min, 2 x/week for 8 weeks, n=15

Exercise group II (asymptomatic):supervised 60 min, 2x/week for 8 weeks, n=11

Control group III (sympt + asympt.): no intervention, n=16

- ➔ Group I: 16 sessions * 20 € /group individual = 320 €
- ➔ Group II: 16 sessions * 20 €/group individual = 320 €
- ➔ Group III: no costs

Cheung et al. (2014) [4]

Exercise group: Unsupervised at home 32 sessions + supervised 8 sessions, 50 min, n = 18:

Control group: no intervention, n=18

- ➔ EG: 8 sessions * 20 € /group individual = 160 €
- ➔ CG: no costs

Jorge et al. (2015) [5]

Exercise group: unsupervised (12+12=24) sessions, 60 min, n = 29 (training time evaluation is based on the exercise description given in the article)

Control group: no intervention, n=31

- ➔ EG (6 weeks period): 12 sessions * 20 € /group individual = 240 €
- ➔ EG (12 weeks period): 24 sessions * 20 € /group individual = 480 €
- ➔ CG: no costs

Topp et al. (2002) [6]

Exercise group I: unsupervised at home 32 session + supervised 16 sessions, 50 min, n=32

Exercise group II: unsupervised at home 32 session + supervised 16 sessions, 50 min, n=35

Control group III: no intervention, n=35

- ➔ Group I: 16 sessions * 15 € /group individual = 240 €
- ➔ Group II: 16 sessions * 15 €/group individual = 240 €
- ➔ Group III: no costs

Fransen et al. (2001) [7] (second baseline assessment not included)

Individual treatment group I: individual 30 min treatment, average 7 sessions, n=43

Exercise group II: supervised 60 min, 2x/week for 8 weeks, n=40

Control group III: no intervention, n=43

- ➔ Group I: 7 sessions * 50 € individual = 350 €
- ➔ Group II: 16 sessions * 20 €/group individual = 320 €
- ➔ Group III: no costs

Wallis et al. (2017) [8]

Exercise group: Individual planning 30 min + individual physiotherapy supervision and monitoring for 12 weeks, n = 23:

Control group: no intervention, n=23

- ➔ EG: 50 € / individual + 12 weeks * 10 € = 170 €
- ➔ CG: no costs

Salacinksi et al. (2012) [9]

Exercise group: At least 2 supervised group cycling for 12 weeks, length of session 60 min, number of trainees in the exercise group, n = 19:

Control group: no intervention, n=18

- ➔ EG: Average 22 cycling sessions * 20 € /group individual = 440 €
- ➔ CG: no costs

Wortley et al. (2013) [10]

Exercise group I: Supervised resistance training 60 min 2x/week for 10 weeks, n=13

Exercise group II: Supervised Tai Ji program 60 min 2x/week for 10 weeks, n=12

Control group III: no intervention, one telephone contact during 10 weeks, n=6

- ➔ Group I: 20 sessions * 20 € /group individual = 400 €
- ➔ Group II: 20 sessions * 20 €/group individual = 400 €
- ➔ Group III: one phone contact per controls * 10 € = 10 €
  (The training sessions were conducted at Senior Center facilities)

O'Reilly et al. (1999) [11]

Exercise group: Unsupervised daily exercise at home for 6 months (~180 sessions). In addition, 3 individual visits to the metrologist (~30 min), n = 113

Control group: no intervention, n=78

- ➔ EG: training no costs. Metrologist: 3 visits * 50 € individual = 150 €
- ➔ CG: no costs

Simao et al. (2012) [12] – contacted to Ana Cristina Lacerda (lacerdaacr@gmail.com)

Exercise group I: Supervised squat exercises in the platform 15 min 3x/week for 12 weeks, n=11

Exercise group II: Supervised squat exercises 15 min 3x/week for 12 weeks, n=10

Control group III: weekly phone calls to each group member, n=11

- ➔ Group I: 36 sessions * 5 € /group individual = 180 €
- ➔ Group II: 36 sessions * 5 €/group individual = 180 €
- ➔ Group III: 12 weeks * 10 € individual = 120 €

Lee et al. (2009) [13]

Exercise group: Supervised mind-body exercise with 60 min sessions 2x/week for 8 weeks, n = 29

Control group: no intervention, n=15

- ➔ EG: 16 sessions * 20 € /group individual = 320 €
- ➔ CG: no costs

Lin et al. (2009) [14]

Exercise group I: Supervised proprioceptive training 50 min 3x/week for 8 weeks, n=36

Exercise group II: Supervised strength training 30 min 3x/week for 8 weeks, n=36 (training time evaluation is based on the exercise description given in the article)

Control group III: no intervention, n=36

- ➔ Group I: 24 sessions * 20 € /group individual = 480 €
- ➔ Group II: 24 sessions * 10 €/group individual = 240 €
- ➔ Group III: no costs

Lim et al. (2008) [15] – contacted to Kim Bennell (k.bennell@unimelb.edu.au)

Exercise malalignment group I: Home exercise 30 - 45 min 5x/week for 12 weeks + 7 individual physiotherapy consultations, n=26

Exercise neutrally aligned group II: Home exercise 30 - 45 min 5x/week for 12 weeks + 7 individual physiotherapy consultations, n=27

Control malalignment group III: no intervention, n=26

Control neutrally aligned group IV: no intervention, n=28

- ➔ Group I: 7 consultations * 75 € individual = 525 €
- ➔ Group II: 7 consultation * 75 € individual = 525 €
- ➔ Group III: no costs
- ➔ Groups IV: no costs

Aglamis et al. (2008) [16]

Exercise group: Supervised multicomponent exercise with 60 min sessions 3x/week for 12 weeks, n = 17

Control group: no intervention, n=14

- ➔ EG: 36 sessions * 20 € /group individual = 720 €
- ➔ CG: no costs

Lee et al. (2008) [17]

Exercise group: Supervised mind-body exercise with 60 min sessions 2x/week for 12 weeks, n = 21

Control group: no intervention, n=15

- ➔ EG: 24 sessions * 20 € /group individual * 21 trainees = 480 €
- ➔ CG: no costs

Rooij et al. (2017) [18]

Exercise group: Supervised individualized comorbidity adapted group exercise program with 30-60 min sessions 2x/week for 20 weeks, n = 63

Control group: no intervention, n=63

- ➔ EG (10 weeks period): 20 sessions * 15 € /group individual = 300 €
- ➔ EG (20 weeks period): 40 sessions * 15 € /group individual = 600 €
- ➔ CG: no costs

Jan et al. (2008) [19]

Exercise group I: Individually supervised high-resistance 30 min 3x/week for 8 weeks, n=34

Exercise group II: Individually supervised low-resistance 50 min 3x/week for 8 weeks, n=34

Control group III: no intervention, n=34

- ➔ Group I: 24 sessions * 50 € individual = 1200 €
- ➔ Group II: 24 sessions * 75 € individual = 1800 €
- ➔ Group III: no costs

Evcik et al. (2002) [20]

Exercise group I: Unsupervised home-based exercise 3x/week for 12 weeks + weekly telephone contact, n=27

Exercise group II: Unsupervised walking program 10-30 min 3x/week for 12 weeks+ weekly telephone contact, n=28

Control group III: no intervention, n=26

- ➔ Group I: 12 telephone contact * 10 € = 120 €
- ➔ Group II: 12 telephone contact * 10 € = 120 €
- ➔ Group III: no costs

Krasilschnikov et al. (2011) [21]

Exercise group: Combined resistance and aerobic exercise program with 60 min sessions 3x/week for 8 weeks, n=8

Control group: no intervention, n=8

- ➔ EG: 24 sessions * 20 € /group individual = 480 €
- ➔ CG: no costs

References

[1] An B, Dai K, Zhu Z, Wang Y, Hao Y, Tang T, et al. Baduanjin alleviates the symptoms of knee osteoarthritis. The Journal of Alternative and Complementary Medicine, 14(2):167–174, 2008.

[2] Bennell KL, Hunt MA, Wrigley TV, Hunter DJ, McManus FJ, Hodges PW, et al. Hip strengthening reduces symptoms but not knee load in people with medial knee osteoarthritis and varus malalignment: a randomised controlled trial. Osteoarthritis and Cartilage, 18(5):621–628, 2010.

[3] Braghin RMB, Libardi EC, Junqueira C, Nogueira-Barbosa MH, and de Abreu DCC. Exercise on balance and function for knee osteoarthritis: a randomized controlled trial. Journal of Bodywork and Movement Therapies, 22(1):76–82, 2018.

[4] Cheung C, Wyman JF, Resnick B, and Savik K. Yoga for managing knee osteoarthritis in older women: a pilot randomized controlled trial. BMC Complementary and Alternative Medicine, 14(1):160, 2014.

[5] Jorge RTB, de Souza MC, Chiari A, Jones A, Fernandes ARC, Júnior IL, et al. Progressive resistance exercise in women with osteoarthritis of the knee: a randomized controlled trial. Clinical Rehabilitation, 29(3):234–243, 2015.

[6] Topp R, Woolley S, Hornyak III J, Khuder S, and Kahaleh B. The effect of dynamic versus isometric resistance training on pain and functioning among adults with osteoarthritis of the knee. Archives of Physical Medicine and Rehabilitation, 83(9):1187–1195, 2002.

[7] Fransen M, Crosbie J, and Edmonds J. Physical therapy is effective for patients with osteoarthritis of the knee: a randomized controlled clinical trial. The Journal of Rheumatology, 28(1):156–164, 2001.

[8] Wallis JA, Webster KE, Levinger P, Singh PJ, Fong C, and Taylor NF. A walking program for people with severe knee osteoarthritis did not reduce pain but may have benefits for cardiovascular health: a phase II randomised controlled trial. Osteoarthritis and Cartilage, 25(12):1969–1979, 2017.

[9] Salacinski AJ, Krohn K, Lewis SF, Holland ML, Ireland K, and Marchetti G. The effects of group cycling on gait and pain-related disability in individuals with mild-to-moderate knee osteoarthritis: a randomized controlled trial. Journal of Orthopaedic & Sports Physical Therapy, 42(12):985–995, 2012.

[10] Wortley M, Zhang S, Paquette M, Byrd E, Baumgartner L, Klipple G, et al. Effects of resistance and Tai Ji training on mobility and symptoms in knee osteoarthritis patients. Journal of Sport and Health Science, 2(4):209–214, 2013.

[11] O'Reilly SC, Muir KR, and Doherty M. Effectiveness of home exercise on pain and disability from osteoarthritis of the knee: a randomised controlled trial. Annals of the rheumatic diseases, 58(1):15–19, 1999.

[12] Simão AP, Avelar NC, Tossige-Gomes R, Neves CD, Mendonça VA, Miranda AS, et al. Functional performance and inflammatory cytokines after squat exercises and whole-body vibration in elderly individuals with knee osteoarthritis. Archives of Physical Medicine and Rehabilitation, 93(10):1692–1700, 2012.

[13] Lee HJ, Park HJ, Chae Y, Kim SY, Kim SN, Kim ST, et al. Tai Chi Qigong for the quality of life of patients with knee osteoarthritis: a pilot, randomized, waiting list controlled trial. Clinical Rehabilitation, 23(6):504–511, 2009.

[14] Lin DH, Lin CHJ, Lin YF, and Jan MH. Efficacy of 2 non-weight-bearing interventions, proprioception training versus strength training, for patients with knee osteoarthritis: a randomized clinical trial. Journal of Orthopaedic & Sports Physical Therapy, 39(6):450–457, 2009.

[15] Lim BW, Hinman RS, Wrigley TV, Sharma L, and L Bennell K. Does knee malalignment mediate the effects of quadriceps strengthening on knee adduction moment, pain, and function in medial knee osteoarthritis? a randomized controlled trial. Arthritis Care & Research: Official Journal of the American College of Rheumatology, 59(7):943–951, 2008.

[16] Aglamış B, Toraman NF, and Yaman H. The effect of a 12-week supervised multicomponent exercise program on knee OA in Turkish women Journal of Back and Musculoskeletal Rehabilitation, 21(2):121–128, 2008.

[17] Lee HY and Lee KJ. Effects of tai chi exercise in elderly with knee osteoarthritis. Journal of Korean Academy of Nursing, 38(1):11–18, 2008.

[18] de Rooij M, van der Leeden M, Cheung J, van der Esch M, Häkki-nen A, Haverkamp D, et al. Efficacy of tailored exercise therapy on physical functioning in patients with knee osteoarthritis and comorbidity: a randomized controlled trial. Arthritis Care & Research, 69(6):807–816,2017.

[19] Jan MH, Lin JJ, Liau JJ, Lin YF, and Lin DH. Investigation of clinical effects of high-and low-resistance training for patients with knee osteoarthritis: a randomized controlled trial. Physical Therapy, 88(4):427–436, 2008.

[20] Evcik D and Sonel B. Effectiveness of a home-based exercise therapy and walking program on osteoarthritis of the knee. Rheumatology International, 22(3):103–106, 2002.

[21] Krasilshchikov O, Shaw I, Sungkit NB, Shaw BS, and Shihabudin TM. Effects of an eight-week training programme on pain relief and physical condition of overweight and obese women with early stage primary knee osteoarthritis: physical activity, health and wellness. African Journal for Physical Health Education, Recreation and Dance, 17(2):328–339, 2011.
